# The effects of nicotine exposure on depression: An integrative analysis combining network toxicology, molecular docking, and Mendelian randomization

**DOI:** 10.18332/tid/220849

**Published:** 2026-06-27

**Authors:** Daoyuan Li, Yuli Huang, Tanyue Zhou, Zhen Wang, Cailing Ren

**Affiliations:** 1 School of Rehabilitation, Gannan Medical University, GanZhou City, China

**Keywords:** nicotine, depression, molecular docking, Mendelian randomization, network toxicology

## Abstract

**INTRODUCTION:**

Depression is a globally prevalent affective disorder closely associated with environmental factors and neurological dysfunction, affecting over 300 million people worldwide and imposing a heavy burden on families and healthcare systems. As the core addictive bioactive component in tobacco, nicotine is intimately linked to an increased risk of depression, but its underlying molecular mechanisms remain unclear. This study aimed to explore the molecular mechanisms of nicotine-induced depression and identify the core molecular targets and pathways involved.

**METHODS:**

This study integrated network toxicology, molecular docking, and Mendelian randomization (MR). Nicotine’s structure and SMILES were retrieved from PubChem for toxicity analysis via ADMETlab 3.0 and Protox3.0. Potential targets of nicotine and depression-related genes were screened from databases including GeneCards, OMIM, and ChEMBL. Common targets were obtained via Venny for PPI network construction, core target screening, GO/KEGG enrichment analysis, and molecular docking of the top 5 targets. MR verified causal associations with depression.

**RESULTS:**

Nicotine exhibited significant neurotoxicity (probability=0.951), respiratory, hepatotoxic, and nephrotoxic effects. A total of 69 common targets were identified, including DRD2, CHRNA4, and STAT3. GO enrichment involved biological processes like excitatory postsynaptic potential and KEGG pathways such as cAMP signaling. Molecular docking showed favorable binding affinity (binding energies < -5.0 kcal/mol). MR confirmed that higher CHRNA4 expression in the Nucleus Accumbens increased depression risk (IVW, OR=1.02; 95% CI: 1.01–1.04, p=0.003).

**CONCLUSIONS:**

This study demonstrated that nicotine exacerbates depressive-like behaviors by binding to core targets, including DRD2 and CHRNA4, and regulating dopaminergic and cholinergic pathways. These findings provide mechanistic insights for elucidating the underlying molecular mechanisms of nicotine-induced depression, while the identified targets and pathways offer potential directions for subsequent mechanistic validation and targeted studies of depression associated with nicotine exposure.

## INTRODUCTION

Depression is a prevalent emotional disorder with high rates of disability and mortality worldwide, whose pathogenesis is closely associated with genetic susceptibility, environmental factors, and neurological dysfunction^1,2^. Epidemiological surveys have indicated that the global population of patients with depression has exceeded 300 million to date, and the disease exhibits a high incidence in both adolescent and adult populations, with the morbidity rate rising year by year among adolescents^3,4^. As a chronic disease, the core pathological characteristics of depression include persistent depressed mood, cognitive impairment, and diminished volitional activity, with clinical manifestations dominated by anhedonia, sleep disturbance, inattention, and other symptoms. It not only severely impairs patients’ quality of life and social functioning but also leads to severe consequences such as self-harm and suicide, imposing a heavy burden on patients’ families and the social medical system^5^. Notably, with the transformation of modern lifestyles, the role of environmental exposure factors in the pathogenesis of depression has gradually become a research focus in academia, among which the pathogenic potential of nicotine, a key bioactive component in tobacco smoke, urgently needs in-depth elucidation^6^.

Nicotine is the core addictive and bioactive substance in tobacco, which is widely present in environmental media such as tobacco smoke and electronic cigarette aerosols. It can easily enter the human body through the respiratory tract, skin, and mucous membranes and distribute rapidly throughout the body, with a particularly high affinity for the central nervous system (CNS)^7^. Nicotine has been proven to exert complex neurotoxic effects, and traditional studies have mostly focused on its addictive mechanisms. In recent years, however, studies have revealed that the toxic effects of nicotine are pleiotropic: in addition to its addictive property, nicotine can disrupt the balance of central neurotransmitters and impair neuronal function, which is closely linked to the occurrence and progression of emotional disorders^8^. Existing epidemiological investigations have demonstrated that the incidence of depression is significantly higher in long-term smokers than in non-smokers, and the intensity of nicotine exposure is positively correlated with the severity of depressive symptoms^9^. Animal experiments have also confirmed that nicotine exposure can induce depressive-like behavioral changes in experimental animals, such as reduced spontaneous activity and anhedonia^10^. Nevertheless, the molecular mechanisms underlying nicotine-induced and exacerbated depression have not been fully elucidated, especially its regulatory effects on depression-related signaling pathways at the molecular level, which still require further exploration.

Network toxicology, an emerging discipline integrating systems biology, genomics, and bioinformatics, reveals the molecular network regulatory mechanisms of pollutant-induced diseases holistically by constructing pollutant-target-disease interaction networks, providing a novel perspective for analyzing pollutant-disease associations^11^. Molecular docking, a structure-based computer simulation technique, accurately predicts the binding mode, affinity, and action sites of small-molecule pollutants with disease target proteins, furnishing critical technical support for verifying their direct interaction and screening core pathogenic targets^12^. Mendelian randomization (MR) infers the population-level causal association between exposure and outcome using genetic variants as instrumental variables, which circumvents confounding bias and reverse causality interference^13^. Complementary to network toxicology, where the latter generates mechanistic hypotheses and screens core targets, and MR performs independent causal verification, this combination enhances the reliability of research findings and supports the exploration of core pathways mediating nicotine-induced depression.

Based on the above technical strengths and complementarity, this study investigated the association between nicotine and depression using network toxicology, molecular docking, and MR. Briefly, network toxicology was used to screen potential targets and candidate regulatory pathways, and construct a protein-protein interaction network. Molecular docking verified the binding affinity of nicotine to these core targets, while MR analyzed their relationship with depression. This study preliminarily explores the potential molecular mechanisms of nicotine in depression from a bioinformatics perspective, providing theoretical support and scientific hypotheses for further research.

## METHODS

Supplementary file Figure 1 presents the workflow for investigating the impact of nicotine exposure on depression using an integrated strategy of network toxicology, molecular docking, and Mendelian randomization.

### Preliminary toxicity prediction and target identification of nicotine

In this study, all the databases and tools employed were referenced from previously published literature, which provided a reliable methodological basis for the conduct of our research. First, the chemical structure of nicotine was retrieved from the PubChem database, and its standard SMILES identifier was obtained. Subsequently, the acquired SMILES string (CN1CCC[C@H]1C2=CN=CC=C2) was input into the ADMETlab 3.0 and Protox3.0 platform for preliminary toxicity analysis. To identify the potential molecular targets of nicotine, a screening was then performed across multiple databases, including SwissTargetPrediction, SEA Search Server, SuperPred, and ChEMBL. During the target prediction process, the species parameter was uniformly set to Homo sapiens to ensure the specificity and reliability of the results^14^.

### Acquisition of depression-associated genes

The search term ‘Depression’ was used to conduct separate retrievals in the GeneCards and OMIM databases^15^. In the GeneCards database, targets were screened according to the median relevance score for depression to enhance their reliability and correlation. The screened GeneCards targets were then integrated with the depression-associated targets obtained from the OMIM database into an Excel spreadsheet, where duplicate entries were merged and removed to finally generate a list of disease-related targets for depression.

### Protein-protein interaction (PPI) network analysis and screening of core target proteins

Nicotine-related and depression-associated targets were input into the Venny platform to obtain their intersecting targets. These intersecting targets were subsequently imported into the STRING protein-protein interaction database, with the species set to ‘Homo sapiens’ and the minimum interaction score threshold set to 0.4, thus generating a PPI network of the common targets of nicotine and depression. To further analyze the topological structure of the network and identify core regulatory targets, the Cytoscape 3.10.1 software was used for the visualization of the PPI network. Nodes were ranked by their Degree values to construct the PPI network map of the common targets of nicotine and depression. In addition, the CytoHubba plugin in Cytoscape, combined with the MCODE algorithm, was employed to screen the top core 10 targets, providing a basis for subsequent mechanistic analysis^16^.

### Gene ontology (GO) and Kyoto Encyclopedia of Genes and Genomes (KEGG) pathway enrichment analyses

The intersecting targets were submitted to the DAVID database for functional annotation and pathway enrichment analysis. In the parameter settings, the identifier was selected as ‘OFFICIAL GENE SYMBOL’, the species was set to ‘Homo sapiens’, and all other parameters were kept as default. GO functional enrichment analysis and KEGG pathway enrichment analysis were then performed separately to reveal the enrichment characteristics of the relevant targets in biological processes, cellular components, molecular functions, and signaling pathways. After the completion of the analyses, the enrichment result data were downloaded and further visualized on a bioinformatics platform to generate bar plots and bubble plots, for the intuitive presentation of key functional categories and pathway information^17^.

### Molecular docking

The top 5 core targets by Degree values were selected for molecular docking with nicotine. Nicotine’s 2D structure from PubChem was optimized to 3D in ChemOffice and saved as mol2. High-quality receptor protein structures from RCSB PDB were processed in PyMOL 2.6 (devoid of redundant components) and saved as PDB. AutoDock 1.5.6 completed pre-docking pretreatment; AutoDock Vina performed molecular docking. Optimal conformations were screened, and Discovery Studio 2019/PyMOL 2.6 visualized nicotine-target binding characteristics^18^.

### Mendelian randomization analysis

This study adopted a two-sample MR approach, using genome-wide association study (GWAS) summary statistics and quantitative trait loci (QTL) data, to explore the causal relationship between drug targets and depression. The validity of MR relies on three core assumptions: 1) relevance, meaning the genetic variants are strongly associated with the exposure; 2) independence, meaning the variants are independent of confounders; and 3) exclusion restriction, meaning the variants affect the outcome solely through the exposure rather than alternative pathways^19^.

To satisfy the relevance assumption, we selected cis-expression quantitative trait loci (cis-eQTLs, ±500 kb) robustly associated with the targets (p<5×10^-5^). Instrumental variables (IVs) for STAT3 were derived from the eQTLGen Consortium (N=31684), while those for CHRNA4, DRD2, MAOB, and SLC6A3 were obtained from depression-relevant brain tissues in the GTEx Project Version 8. MAOB and SLC6A3 were subsequently excluded due to the lack of valid eQTLs meeting this threshold. Furthermore, we calculated F-statistics for each variant, retaining only those with F ≥10 to further ensure instrument relevance and minimize weak instrument bias. To address the independence assumption, linkage disequilibrium (LD) clumping (r^2^<0.1, window=500 kb) was performed to ensure that the selected single nucleotide polymorphisms (SNPs) were statistically independent^20^.

The primary MR analysis employed the inverse variance weighted (IVW) method for targets with multiple IVs (CHRNA4, STAT3) and the Wald ratio method for targets with a single IV (DRD2). Crucially, to evaluate the exclusion restriction assumption, multiple sensitivity analyses were conducted for targets with ≥3 SNPs. Specifically, Cochran’s Q statistic was used to assess heterogeneity among IVs, while the MR-Egger intercept test and the MR-PRESSO (Pleiotropy RESidual Sum and Outlier) global test were employed to rigorously detect and correct for horizontal pleiotropy. These steps ensured that the IVs did not affect the risk of depression through confounding pleiotropic pathways. All analyses were performed using the *TwoSampleMR* and *MRPRESSO* packages in R software.

## RESULTS

### Toxicity prediction of nicotine

Based on the toxicity prediction results from the ADMETlab 3.0 and ProTox3.0 databases (Table 1; and Supplementary file Figure 2), nicotine exhibited prominent neurotoxicity with a probability of 0.951, along with respiratory, hepatotoxic, and nephrotoxic effects. Given the aforementioned toxicological characteristics, this study further focused on the potential role of nicotine in inducing chronic neurological disorders such as depression, aiming to identify the associated targets and molecular mechanisms.

**Table 1:** Toxicity prediction of nicotine

*Database*	*Toxicity*	*Probability*
ADMETlab3.0	Neurotoxicity	0.951
	Hepatotoxicity	0.844
	Nephrotoxicity	0.815
ProTox3.0	Respiratory Toxicity	0.73 (Active)
	Neurotoxicity	0.67 (Active)
	Ecotoxicity	0.60 (Active)

### Prediction of intersecting targets of nicotine and depression

A total of 23, 25, 110, and 140 potential targets of nicotine were predicted using SwissTargetPrediction, SEA, ChEMBL, and SuperPred databases, respectively. After standardization and duplicate removal, 217 nicotine-associated targets were obtained. Meanwhile, 1978 highly depression-related targets were screened from the GeneCards database via median filtering, and 35 relevant genes were identified from the OMIM database. Following integration and duplicate elimination, a total of 1980 targets highly associated with depression were acquired. Venn diagram analysis of the compound and disease targets finally identified 69 intersecting targets (Supplementary file Figure 3).

### Identification of core targets via PPI network analysis

The constructed PPI network consisted of 69 nodes and 272 interaction edges. The color depth of nodes was positively correlated with their degree values, darker colors indicated higher degree values and greater centrality in the entire network, suggesting that the corresponding targets exerted more critical core roles in the network (Supplementary file Figure 4). Further in-depth analysis of the PPI network identified the top 10 core targets, which were DRD2, CHRNA4, STAT3, MAOB, SLC6A3, SLC6A4, GABRA1, CHRNB2, DRD1 and MAOA in descending order (Table 2; and Supplementary file Figure 5). These results indicated that these core target genes might play pivotal roles in the molecular regulatory mechanisms underlying nicotine-induced depression.

**Table 2:** Top ten core targets according to degrees of freedom

*Core targets*	*Degree*	*Closeness centrality*	*Betweenness centrality*
DRD2	23	0.55737705	0.16444998
CHRNA4	21	0.48920863	0.08417899
STAT3	20	0.50746269	0.16926139
MAOB	19	0.53543307	0.11115121
SLC6A3	17	0.47552448	0.03490350
SLC6A4	16	0.45637584	0.02119158
GABRA1	15	0.46896552	0.04324802
CHRNB2	15	0.42767296	0.02279099
DRD1	15	0.45333333	0.01476656
MAOA	15	0.48571429	0.05860032

### GO and KEGG pathway profiles of potential targets

To explore the potential biological functions of the intersecting targets involved in nicotine-induced depression, GO and KEGG pathway enrichment analyses were performed in this study. GO analysis included three categories: biological process (BP), cellular component (CC), and molecular function (MF). The BP category was mainly involved in excitatory postsynaptic potential, membrane potential regulation, and chemical synaptic transmission; the CC category was enriched in structures such as monatomic ion channels and acetylcholinegated monatomic cation-selective channels; the MF category was dominated by response to nicotine, acetylcholine binding, and homophilic protein binding. The top 10 significant terms of the above categories were visualized (Supplementary file Figure 6A). KEGG pathway enrichment analysis revealed that cocaine addiction, serotonergic synapse, and neurodegeneration-multiple diseases pathways were highly enriched (Supplementary file Figure 6B).

### Molecular docking

The 2D molecular structure of nicotine was retrieved from the PubChem database, while the top five potential core targets (DRD2, CHRNA4, STAT3, MAOB, and SLC6A3) and their crystal structures were obtained from the PDB database with the corresponding PDB IDs: 5AER, 5KXL, 8A27, 6FW0, and 9EO4, respectively. Molecular docking results demonstrated that nicotine exhibited favorable binding affinities to all five core targets. According to the binding energy evaluation criterion, a value of <-5.0 kcal/mol indicates a good binding interaction, and a lower value represents a stronger affinity and conformational stability. The binding energies of nicotine to DRD2, CHRNA4, STAT3, MAOB, and SLC6A3 were -5.4, -5.1, -6.9, -6.5, and -6.8 kcal/ mol, respectively, indicating that nicotine had a high affinity for these targets (Table 3; and Supplementary file Figure 7).

**Table 3:** Molecular docking binding free energies of nicotine to depression target proteins

*Targets*	*Uniprot ID*	*PDB ID*	*Binding energy (kcal/mol)*
DRD2	P14416	5AER	-5.4
CHRNA4	P43681	5KXL	-5.1
STAT3	P00533	8A27	-6.9
MAOB	P27388	6FW0	-6.5
SLC6A3	P01959	9EO4	-6.8

### MR reveals the impact of core targets on depression

After strict quality control, MAOB was excluded due to insufficient qualified cis-eQTLs, leaving CHRNA4 (5 SNPs), DRD2 (1 SNP), and STAT3 (15 SNPs) for MR analysis. All retained SNPs possessed *F*-statistics >10, satisfying the relevance assumption by minimizing weak instrument bias. Genetically predicted higher CHRNA4 expression in the Nucleus Accumbens was associated with a modest but statistically significant increase in depression risk (IVW, OR=1.02; 95% CI: 1.01–1.04, p=0.003), with consistent directions across sensitivity analyses. Conversely, higher DRD2 expression in the Anterior Cingulate Cortex significantly reduced depression risk (Wald ratio, OR=0.95; 95% CI: 0.91–0.99, p=0.007). No causal relationship was found between STAT3 expression and depression (IVW, OR=1.00; p=0.95). Crucially, evaluating the exclusion restriction assumption, sensitivity analyses showed no significant directional pleiotropy (MR-Egger intercept p>0.05). While some heterogeneity was observed for STAT3, this was accounted for by the random-effects model, confirming the overall robustness of the causal estimates (Supplementary file Figure 8).

## DISCUSSION

This study integrated network toxicology, molecular docking, and Mendelian randomization techniques to systematically elucidate the core molecular mechanisms underlying nicotine-induced depression. As the key addictive component of tobacco, nicotine features high liposolubility and ready blood-brain barrier penetration; upon inhalation, it rapidly accumulates in emotion-regulating brain regions such as the hippocampus. Long-term nicotine exposure is closely associated with an elevated risk of depression, exacerbated depressive symptoms, and treatment resistance, rendering the mechanistic elucidation of this association of great clinical significance^21^. Existing studies have confirmed that nicotine disrupts the homeostasis of the central nervous system through two distinct pathways: first, it interferes with the balance of synthesis and reuptake of key neurotransmitters including dopamine and serotonin, thus impairing the neurochemical foundation of emotional regulation; second, it triggers microglia-mediated neuroinflammation, promotes the release of proinflammatory cytokines such as IL-6 and TNF-α, induces neuronal damage and reduced synaptic plasticity, and ultimately leads to the dysregulation of emotional regulation^22,23^.

This study confirmed that nicotine exhibits a high binding affinity for core targets, including DRD2, CHRNA4, and STAT3. It can form a multi-target and multi-pathway regulatory network by modulating neurotransmitter transmission as well as the dopaminergic and cholinergic pathways, thereby exacerbating depressive-like behaviors.

DRD2 is a target of the dopamine reward and emotional regulation pathway, and its genetic polymorphism can affect an individual’s susceptibility to nicotine dependence by regulating receptor function and the sensitivity of the dopamine system. Nicotine exposure can upregulate DRD2 expression or enhance its signal transduction, alter dopamine levels and neuroplasticity in emotion-related brain regions such as the prefrontal cortex and hippocampus, and thereby participate in mediating the onset of depression^24^. A study by Yang et al.^25^ demonstrated that nicotine exposure can also enhance the neuroinflammatory response by modulating DRD2 expression and the downstream β-arrestin2/STAT3 signaling axis, further aggravating the pathological process of depression; conversely, biased agonists targeting DRD2 exert antidepressant effects by restoring pathway homeostasis^25^. The molecular docking results of this study showed that the binding energy of nicotine to DRD2 was -5.4 kcal/mol, indicating a high-affinity interaction. This suggests that nicotine may regulate the pathological process of depression by acting on DRD2.

CHRNA4 is mainly localized in key brain regions for emotional regulation, including the hippocampus and prefrontal cortex. It modulates neuronal excitability, maintains synaptic plasticity, and regulates neurotransmitter release; its functional imbalance disrupts the synergistic effect of multiple neurotransmitter systems, which is an important pathological link in the onset of depression^26^. The validation results of this study showed that the binding energy of nicotine to CHRNA4 reached -5.1 kcal/mol, indicating favorable binding activity between the two. Based on this, we speculate that nicotine may disrupt the homeostasis of the cholinergic system by acting on CHRNA4, thereby mediating the onset of depression.

STAT3 is a multifunctional transcription factor abundantly expressed in neurons and glia. Its aberrantly activated neuroinflammation constitutes a core pathological mechanism of depression, triggering emotional dysregulation via proinflammatory cytokine release and neuronal injury. Zhao et al.^27^ confirmed that the STAT3 pathway in the prefrontal cortex mediates the comorbidity of neuropathic pain and depression, and its abnormal activation exacerbates neuroinflammation and impairs the function of emotion-regulating brain regions. Nicotine exposure can induce neuroinflammation and activate the STAT3 pathway, disrupting brain homeostasis, which suggests that STAT3 is a target linking inflammation and emotional disturbance in nicotine-mediated depression^27^. Clinical studies by Morvarid et al.^28^ also verified that the abnormal activation of STAT3 has important clinical significance in the onset of depression and serves as a crucial regulatory target for its occurrence and development^28^. The molecular docking results of this study showed that nicotine had the highest binding energy to STAT3 (-6.9 kcal/mol) with strong binding activity, suggesting that nicotine may mediate the onset of depression by regulating the transcriptional activity of STAT3.

The monoamine oxidase B encoded by MAOB is a key enzyme in the metabolism of monoamine neurotransmitters, and its functional abnormality disrupts the homeostasis of the brain monoaminergic system and impairs emotional regulation^29^. Nicotine exposure can alter MAOB expression and enzymatic activity, exacerbate the metabolic imbalance of monoamine transmitters, and damage the function of emotion-related brain regions. It can also regulate MAOB gene expression through epigenetic modification, alter the activity of its encoded enzyme, interfere with the metabolic balance of transmitters such as dopamine, disrupt the neurochemical homeostasis of emotional regulation in the brain, and mediate the depressive pathological process^30^. Molecular docking in this study showed that the binding energy of nicotine to MAOB reached -6.5 kcal/mol, indicating a high-affinity combination.

Clinical studies have shown that the C>T single nucleotide polymorphism (SNP) at the rs40184 locus of the SLC6A3 gene is significantly associated with depression. Genetic variation at this locus can serve as a potential genetic risk marker for the onset of depression, and its polymorphic characteristics are involved in regulating the genetic susceptibility to depression, making it one of the important genetically related targets for depression pathogenesis^31^. Animal experiments have also confirmed that abnormal SLC6A3 expression causes dysregulated dopamine reuptake and an imbalance in the brain dopaminergic system, which not only participates in the pathological onset of depression but also mediates the development of depression-related erectile dysfunction, serving as a target for regulating dopaminergic signaling in depressive states^32^. The molecular docking results of this study showed that the binding energy of nicotine to SLC6A3 reached -6.8 kcal/mol with strong binding activity, indicating that SLC6A3 may be a target for nicotine to regulate dopamine homeostasis and induce depression.

KEGG pathway enrichment analysis revealed that the intersecting targets of nicotine exposure and depression were significantly enriched in core pathways including the cAMP signaling pathway, dopaminergic synapse, serotonergic synapse, and cholinergic synapse. These pathways may jointly mediate the pathological process of nicotine-induced depression through specific binding and regulation with core targets such as STAT3, MAOB, DRD2, SLC6A3, and CHRNA4. The cAMP pathway is a key secondary messenger pathway regulating depression; STAT3 can modulate its signal transduction efficiency, thereby affecting neuronal proliferation and neurotransmitter release. Abnormal activation of this pathway leads to functional disorders in emotion-related brain regions, and STAT3-mediated dysregulated regulation of the cAMP pathway is an important molecular mechanism of depression^33^. The dopaminergic synapse pathway is directly involved in the reward mechanism and emotional regulation. Binding of nicotine to DRD2, a dopamine receptor subtype, can inhibit the activity of dopaminergic neurons, while SLC6A3-mediated abnormal enhancement of dopamine reuptake jointly leads to decreased brain dopamine levels, inducing core depressive symptoms such as anhedonia and depressed mood, which is consistent with the clinical characteristics of abnormal dopaminergic system function in patients with depression^34^. In the serotonergic synapse pathway, MAOB, a subtype of monoamine oxidase, can accelerate serotonin degradation upon abnormal activation; nicotine may further reduce serotonin levels by upregulating MAOB expression, exacerbating emotional regulation dysfunction^35^. The enrichment of the cholinergic synapse pathway is closely related to the regulation of CHRNA4; nicotine may further exacerbate neural circuit imbalance by activating CHRNA4-mediated cholinergic neurotransmission, promoting the formation of depressive-like phenotypes^36^. Consequently, by binding to core targets with high affinity, nicotine synchronously activates the synergistic effect of multiple pathways, disrupts CNS homeostasis, and ultimately induces depression. This provides a critical target-pathway level theoretical basis for elucidating the molecular mechanism of nicotine-induced depression and developing targeted interventions.

MR analysis provided crucial genetic evidence to validate the molecular pathways identified by network pharmacology and molecular docking. Notably, cis-eQTLs were used to proxy the lifelong expression of target genes (e.g. CHRNA4 and DRD2) instead of directly measuring environmental nicotine exposure. The significant causal associations – CHRNA4 overexpression increasing depression risk and DRD2 expression exerting a protective effect – confirmed these targets’ pathogenic relevance, bridging network toxicology and genetics and suggesting nicotine-induced depression may be partially mediated by these genetically validated receptor pathways. Although the effects were statistically robust, effect sizes were modest (e.g. OR=1.02 for CHRNA4), a recognized feature of cis-eQTL-based drug-target MR studies. These effect sizes reflect causal direction and target validity, not direct quantification of smoking-related clinical risk, with our integrated analysis highlighting pathway relevance and adopting a cautious interpretation of macroscopic environmental impacts.

Analyzing the effects of nicotine on depression by integrating network toxicology, molecular docking, and Mendelian randomization offers distinct and complementary advantages, providing solid support for relevant research: first, it constructs pollutant-disease molecular associations, mines core targets and pathways by integrating multiple databases, and avoids the limitations of single-target studies. Meanwhile, Mendelian randomization relies on genetic data to effectively exclude confounding bias, clarify associations, and avoid reverse causality interference; second, molecular docking quantifies target binding capacity and visualizes experimental results, facilitating intuitive understanding of mechanisms, while Mendelian randomization verifies the genetic correlation between targets and diseases, enhancing the reliability of the proposed mechanisms; third, it clarifies precise research directions for subsequent experiments, reduces research blindness, locates key pathological links, and provides a theoretical basis for depression prevention, control, and intervention research.

### Limitations

This study has several limitations, and the conclusions are only mechanism hypotheses based on dry experiments, which have not been verified by *in vitro*, *in vivo*, and clinical studies; their scientific robustness and applicability need further confirmation by subsequent research. First, the research methods have inherent limitations: all results were obtained through network toxicology database analysis and *in vitro* molecular docking simulation, lacking direct verification by *in vitro* cell experiments and *in vivo* animal experiments. The associations between some targets and pathways in public databases are insufficiently supported by experimental evidence, which renders the *in vivo* reliability and applicability of the research conclusions to be further confirmed. Second, the study design did not clarify the dose-effect and time-effect relationships of nicotine exposure, nor did it explore the associations between different nicotine exposure doses, exposure durations, depression risk, and core target/pathway activation levels, making it difficult to truly reflect the toxicological effects of nicotine exposure in complex real environments. Third, the technical methods have inherent defects; the completeness of the databases used and the accuracy of the analysis algorithms may affect the accuracy of the results, which may lead to the omission of some key pathogenic mechanisms.

## CONCLUSIONS

This study integrated network toxicology, molecular docking, and Mendelian randomization approaches to identify potential core targets (e.g. DRD2, CHRNA4) and related regulatory pathways involved in nicotine-induced depression, which fully leverages the advantages of multi-technical integration and effectively compensates for the limitations of single research methods. These core targets and pathways provide verifiable potential biomarkers and intervention targets for depression associated with nicotine exposure, and offer exploratory directions for subsequent *in vivo* experimental validation and clinical translational research.

## Supplementary Material



## Data Availability

The data supporting this research are available from the sources listed in the Supplementary file.

## References

[1] Tian X, Bai FF, Zhao YP, et al. Immediate memory is associated with alexithymia in Chinese Han first-episode, drug-naïve major depressive disorder. Front Psychiatry. 2025;16:1473204. doi:10.3389/fpsyt.2025.1473204 40206647 PMC11978825

[2] Hu L, Wang J, Zhao X, Cai D. Mechanism of saikogenin G against major depressive disorder determined by network pharmacology. Medicine (Baltimore). 2022;101(34):e30193. doi:10.1097/MD.0000000000030193 36042622 PMC9410695

[3] Huang H, Guo Z, Yang X, Qin L. Functional brain alterations in anxious depression: insights from whole-brain fMRI and meta-analysis. Dialogues Clin Neurosci. 2026;28(1):32-45. doi:10.1080/19585969.2026.2612918 PMC12821352 41555810

[4] Hao Y, Yin J, Huang Z, et al. Association between traditional Chinese medicine constitution and depression in adolescents: a cross-sectional study. Medicine (Baltimore). 2026;105(4):e47310. doi:10.1097/MD.0000000000047310 41578533 PMC12851703

[5] Castillo Zorro CA, Fonzo GA, Moscoso-Barrera WD. Use of physiological signals, behavioral data, and processing algorithms in electronic devices and mobile applications for diagnosing depression, anxiety, and stress. Digit Health. 2026;12:20552076251404514. doi:10.1177/20552076251404514 41567417 PMC12816561

[6] Nolder KA, Gaalema DE, Katz BR, et al. Effects of very low nicotine content cigarettes and concurrent provision of e-cigarettes on symptoms of depression and anxiety. Exp Clin Psychopharmacol. 2025;33(6):638-644. doi:10.1037/pha0000803 40965925 PMC12584977

[7] Jiang J, Li X, Hu AF, et al. Nicotine and neuronal nicotinic acetylcholine receptors: unraveling the mechanisms of nicotine addiction. Front Neurosci. 2025;19:1670883. doi:10.3389/fnins.2025.1670883 41179996 PMC12575229

[8] Archie SR, Sharma S, Burks E, Abbruscato T. Biological determinants impact the neurovascular toxicity of nicotine and tobacco smoke: a pharmacokinetic and pharmacodynamics perspective. Neurotoxicology. 2022;89:140-160. doi:10.1016/j.neuro.2022.02.002 35150755 PMC8958572

[9] Xiong H, Ma F, Tang D, Liu D. Correlations among nicotine dependence, health-related quality of life, and depression in current smokers: a cross-sectional study with a mediation model. Front Psychiatry. 2024;15:1455918. doi:10.3389/fpsyt.2024.1455918 39257561 PMC11384568

[10] Pekala K, Michalak A, Kruk-Slomka M, Budzynska B, Biala G. Impacts of cannabinoid receptor ligands on nicotine- and chronic mild stress-induced cognitive and depression-like effects in mice. Behav Brain Res. 2018;347:167-174. doi:10.1016/j.bbr.2018.03.019 29551733

[11] He P, Lu Z, Zhao Y, Li T, Chen J. Integrated network toxicology and molecular simulations uncover the molecular mechanism and core targets of DNBaP-induced lung cancer. Discov Oncol. 2025;16(1):2337. doi:10.1007/s12672-025-04053-2 41310080 PMC12753595

[12] Yao ZY, Fan SY, Song ZF, Li ZC. Network pharmacology-based and molecular docking-based analysis of You-Gui-Yin for the treatment of osteonecrosis of the femoral head. Medicine (Baltimore). 2023;102(43):e35581. doi:10.1097/MD.0000000000035581 37904445 PMC10615424

[13] Dai Z, Wang B, Yin H, Zhang Q. Uncovering the molecular network of nicotine induced erectile dysfunction through network toxicology and mendelian randomization. Reprod Toxicol. 2026;139:109114. doi:10.1016/j.reprotox.2025.109114 41274466

[14] Xu S, Wang G, Liu J, et al. Unraveling the mechanisms of nicotine-induced osteoporosis via network toxicology, bioinformatics, and molecular docking. Tob Induc Dis. 2026;24(January). doi:10.18332/tid/215177 PMC12817853 41568086

[15] Xu K, Zhang Q, Shen Z, Zeng J, Liao Y. Analysis of toxicity and mechanisms of aspartame in kidney stones with network toxicology and molecular docking strategy. Sci Rep. 2025;15(1):45563. doi:10.1038/s41598-025-29822-5 41310176 PMC12749232

[16] Peng C, Wei J, Chen W, Liang F, Huang Z, Huang Y. Analysis of the effects of perfluorooctane sulfonate on COPD using network toxicology and molecular docking. iScience. 2025;28(9):113372. doi:10.1016/j.isci.2025.113372 PMC12454616 40995116

[17] Chen L, Huang YL, Liu F, et al. Impact of Di-(2-Ethylhexyl)-Phthalate on metabolic syndrome: insights from network toxicology and molecular docking and dynamics. Diabetes Metab Syndr Obes. 2025;18:2277-2288. doi:10.2147/DMSO.S523668 40655169 PMC12255270

[18] Mu Y, Zhou Y, Zhang X, Shao Y. Exploring the mechanisms and targets of proton pump inhibitors-induced osteoporosis through network toxicology, molecular docking, and molecular dynamics simulations. Front Pharmacol. 2025;16:1592048. doi:10.3389/fphar.2025.1592048 40421218 PMC12104171

[19] Xu B, Xiang P, Yang X, et al. Explore the potential mechanisms between ertugliflozin and kidney cancer through bioinformatics analysis and Mendelian randomization study. Int J Surg. 2026. doi:10.1097/JS9.0000000000004970 41706624

[20] Gorman BR, Ji SG, Francis M, et al. Multi-ancestry GWAS meta-analyses of lung cancer reveal susceptibility loci and elucidate smoking-independent genetic risk. Nat Commun. 2024;15(1):8629. doi:10.1038/s41467-024-52129-4 39366959 PMC11452618

[21] Fang Y, Yang R, Song R, et al. Nicotine exposure in adolescence triggers the activation and subsequent damage of microglia in the dentate gyrus and promotes depression later in life. Int Immunopharmacol. 2025;161:115060. doi:10.1016/j.intimp.2025.115060 40513337

[22] Liu Y, Zhang L, Fu S, Wei S, Jin Z, He L. Gender differences in the relationship between nicotine exposure and symptoms of depression. Pharmacol Biochem Behav. 2024;244:173857. doi:10.1016/j.pbb.2024.173857 39154790

[23] Amiry GY, Haidary M, Azhdari-Zarmehri H, Beheshti F, Ahmadi-Soleimani SM. Omega-3 fatty acids prevent nicotine withdrawal-induced exacerbation of anxiety and depression by affecting oxidative stress balance, inflammatory response, BDNF and serotonin metabolism in rats. Eur J Pharmacol. 2023;947:175634. doi:10.1016/j.ejphar.2023.175634 36868293

[24] Del Casale A, Paolini M, Gentile G, et al. Dopamine DRD2 and DRD3 polymorphisms involvement in nicotine dependence in patients with treatment-resistant mental disorders. J Pers Med. 2022;12(4):565. doi:10.3390/jpm12040565 35455685 PMC9033085

[25] Liu Y, Song N, Yao H, et al. β-Arrestin2-biased Drd2 agonist UNC9995 alleviates astrocyte inflammatory injury via interaction between β-arrestin2 and STAT3 in mouse model of depression. J Neuroinflammation. 2022;19(1):240. doi:10.1186/s12974-022-02597-6 36183107 PMC9526944

[26] Pan C, Liu J, Gao Y, et al. Hepatocyte CHRNA4 mediates the MASH-promotive effects of immune cell-produced acetylcholine and smoking exposure in mice and humans. Cell Metab. 2023;35(12):2231-2249.e7. doi:10.1016/j.cmet.2023.10.018 38056431

[27] Zhao YT, Deng J, Liu HM, et al. Adaptation of prelimbic cortex mediated by IL-6/STAT3/Acp5 pathway contributes to the comorbidity of neuropathic pain and depression in rats. J Neuroinflammation. 2022;19(1):144. doi:10.1186/s12974-022-02503-0 35690777 PMC9188197

[28] Noormohammadi M, Etesam F, Amini A, Dehkordi PK, Mohammadzadeh M, Shidfar F. Impact of Nano-Selenium supplementation on the JAK/STAT signaling pathway in major depressive disorder: a Triple-Blind, randomized controlled trial. BMC Psychiatry. 2025;25(1):785. doi:10.1186/s12888-025-07213-4 40796838 PMC12341080

[29] Gonzalez I, Polvillo R, Ruiz-Galdon M, Reyes-Engel A, Royo JL. MAOB rs3027452 modifies mood improvement after tryptophan supplementation. Int J Gen Med. 2021;14:1751-1756. doi:10.2147/IJGM.S305443 33986613 PMC8110250

[30] Ziegler C, Domschke K. Epigenetic signature of MAOA and MAOB genes in mental disorders. J Neural Transm (Vienna). 2018;125(11):1581-1588. doi:10.1007/s00702-018-1929-6 30242487

[31] Bumrungthai S, Buddhisa S, Duangjit S, Passorn S, Sumala S, Prakobkaew N. Association of HHV-6 reactivation and SLC6A3 (C>T, rs40184), BDNF (C>T, rs6265), and JARID2 (G>A, rs9383046) single nucleotide polymorphisms in depression. Biomed Rep. 2024;21(6):181. doi:10.3892/br.2024.1869 39420919 PMC11484186

[32] Hong ZM, Chen ZL, Feng JL, et al. Mechanistic analysis of erectile dysfunction in a depression rat model. J Int Med Res. 2022;50(5):3000605221100334. doi:10.1177/03000605221100334 35615771 PMC9152200

[33] Han L, Wei S, Wang R, Liu Y, Zhong Y, Luo H. Exploration of the mechanisms of Acorustatarinowii in the treatment of major depressive disorder based on network pharmacology and molecular docking techniques. Curr Issues Mol Biol. 2025;47(5):342. doi:10.3390/cimb47050342 40699741 PMC12109585

[34] Guo Z, Li S, Wu J, Zhu X, Zhang Y. Maternal deprivation increased vulnerability to depression in adult rats through DRD2 promoter methylation in the ventral tegmental area. Front Psychiatry. 2022;13:827667. doi:10.3389/fpsyt.2022.827667 35308874 PMC8924051

[35] Šimić G, Vukić V, Kopić J, Krsnik Ž, Hof PR. Molecules, mechanisms, and disorders of self-domestication: keys for understanding emotional and social communication from an evolutionary perspective. Biomolecules. 2020;11(1):2. doi:10.3390/biom11010002 33375093 PMC7822183

[36] Gao J, Davis LK, Hart AB, et al. Genome-wide association study of loneliness demonstrates a role for common variation. Neuropsychopharmacology. 2017;42(4):811-821. doi:10.1038/npp.2016.197 27629369 PMC5312064

