# Supplementary material for: The effects of nicotine exposure on depression: An integrative analysis combining network toxicology, molecular docking, and Mendelian randomization
Source: Tob Induc Dis. 2026 Jun 27;24:10.18332/tid/220849. doi: 10.18332/tid/220849 (PMC13312265; doi:10.18332/tid/220849)
Supplement: Supplementary file 1 [file TID-24-99-s1.pdf]

 DOI:10.18332/tid/220849

## Supplementary file

DATA AVAILABILITY

The data supporting this research are available from the following links:

1. PubChem (https://pubchem.ncbi.nlm.nih.gov/accessed on January 10, 2026)

2. ADMETlab3.0 ‹https://admetlab3.scbdd.com/accessed on January 10, 2026›

3. ProTox3.0 (https://tox.charite.de/ accessed on January 10, 2026)

4. SwissTargetPrediction (https://swisstargetprediction.ch/accessed on January 10, 2026)

5. Super Pred (prediction.charite.de/accessed on January10, 2026)

6. SEA Search Server (https://sea.bkslab.org/accessed on January 10, 2026)

7. ChEMBL (https://www.ebi.ac.uk/chembl/accessed on January 10, 2026)

8. GeneCards (https://www.genecards.org/accessed on January11, 2026)

9. OMIM (https://www.omim.org/accessed on January11, 2026)

10. Venny (https://bioinfogp.cnb.csic.es/tools/venny/accessed on January 11, 2026)

11. STRING (https://cn.string-db.org/accessed on January 11, 2026)

12. DAVID (https://davidbioinformatics.nih.gov/accessed on January 11, 2026)

13. The microbiome visualization cloud platform

14. RCSB PDB ‹https://www.rcsb.org/pages/about-us/index/accessed on January 12, 2026›

15. GAWS (https://gwas.mrcieu.ac.uk/accessed on January 13, 2026)

16. eQTL (https://www.eqtlgen.org/accessed on January 13, 2026)

17. GTEX (https://gtexportal.org/accessed on January 13, 2026)

 DOI:10.18332/tid/220849

Figure 1. Research workflow of integrative analysis for nicotine-depression molecular mechanism based on network toxicology, molecular docking and Mendelian randomization

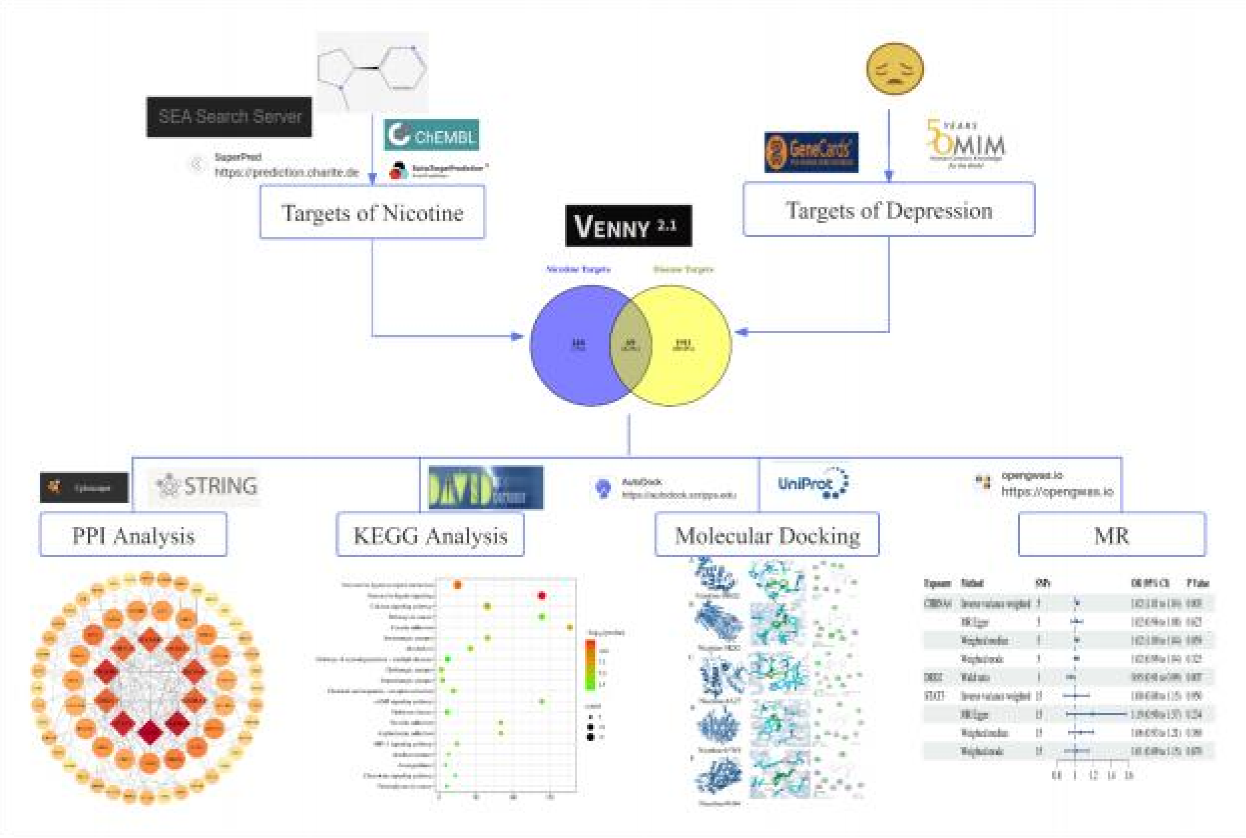

 DOI:10.18332/tid/220849

Figure 2. Potential toxic effect distribution of nicotine from in silico analysis based on ADMETlab 3.0 and ProTox3.0 databases

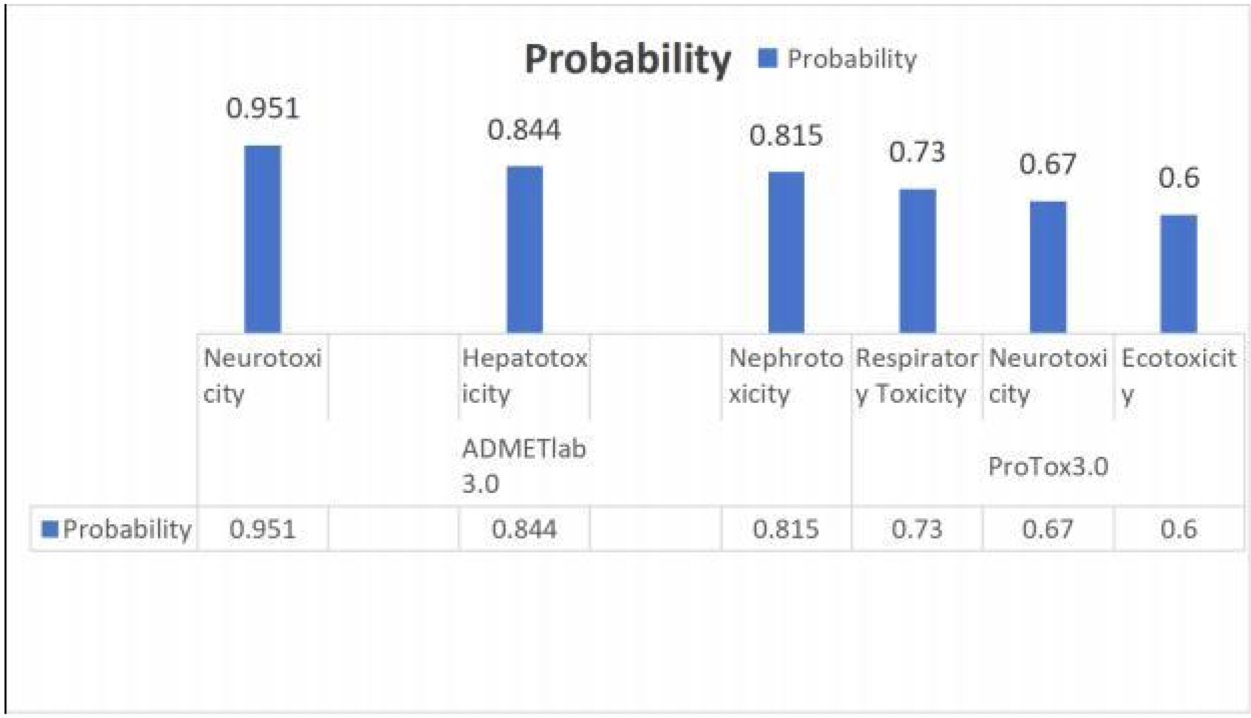


Figure 3. Venn diagram of overlapping targets between nicotine-related genes and depression-related genes

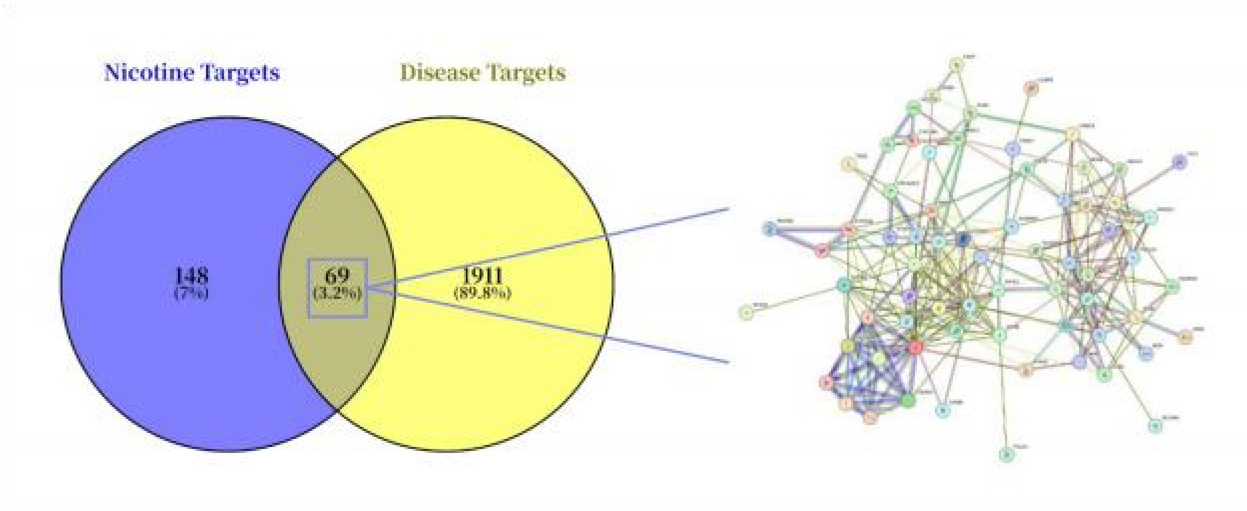

 DOI:10.18332/tid/220849

Figure 4. PPI interaction network of nicotine-depression overlapping targets

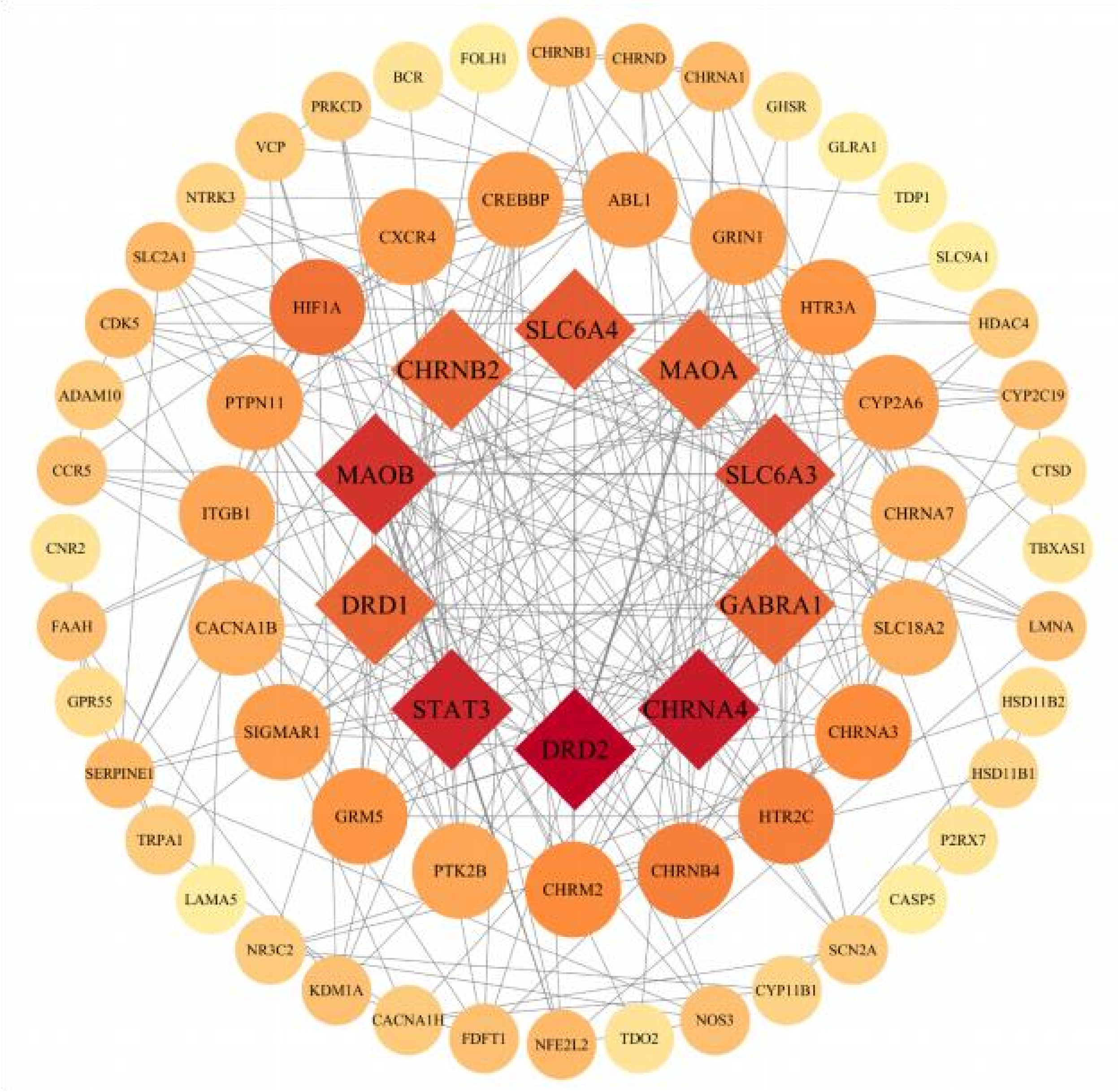

Figure 5. Top 10 core target nodes in nicotine-depression PPI network

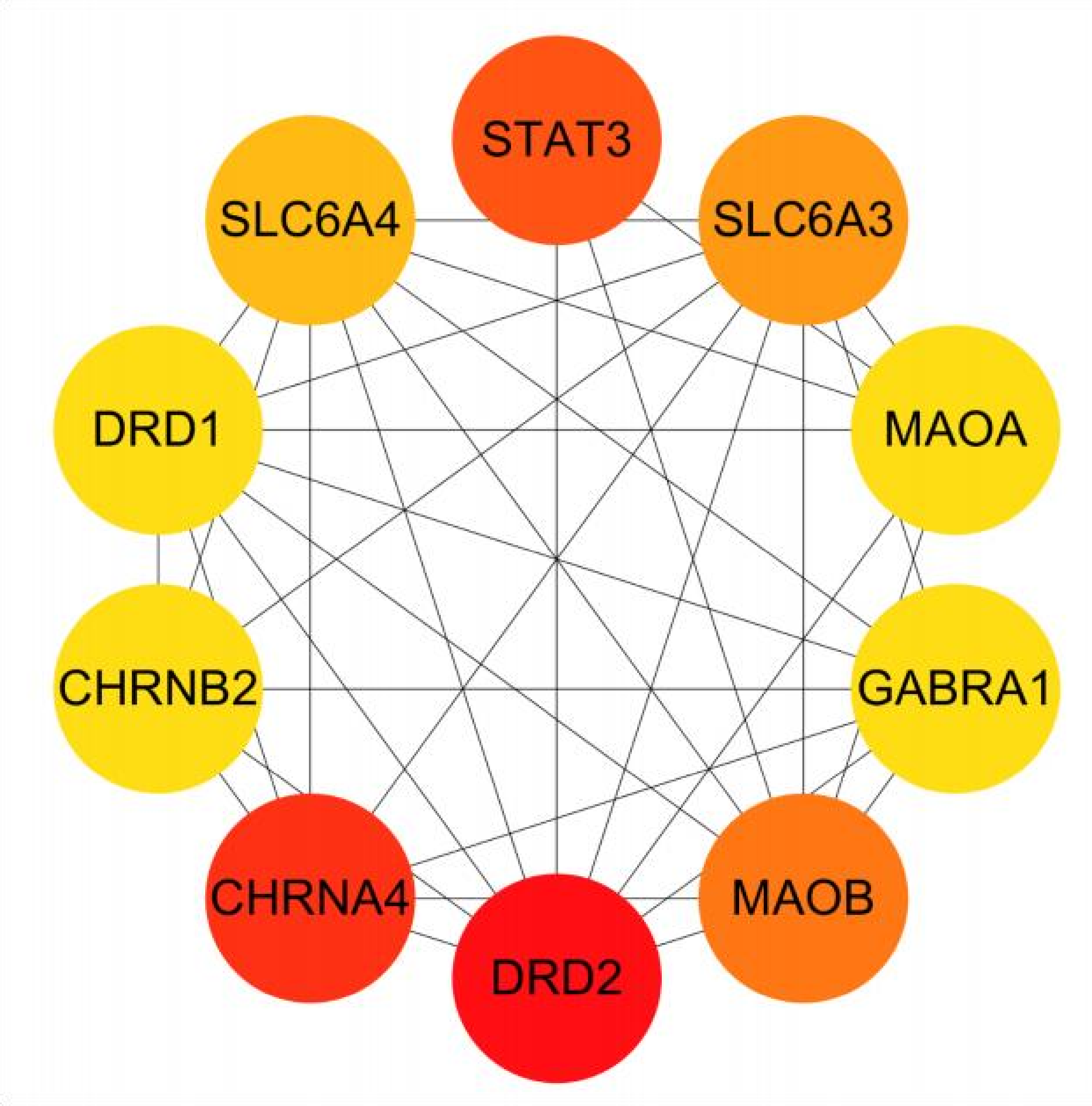

 DOI:10.18332/tid/220849

Figure 6. GO functional enrichment and KEGG pathway enrichment analysis results of nicotine-depression overlapping targets

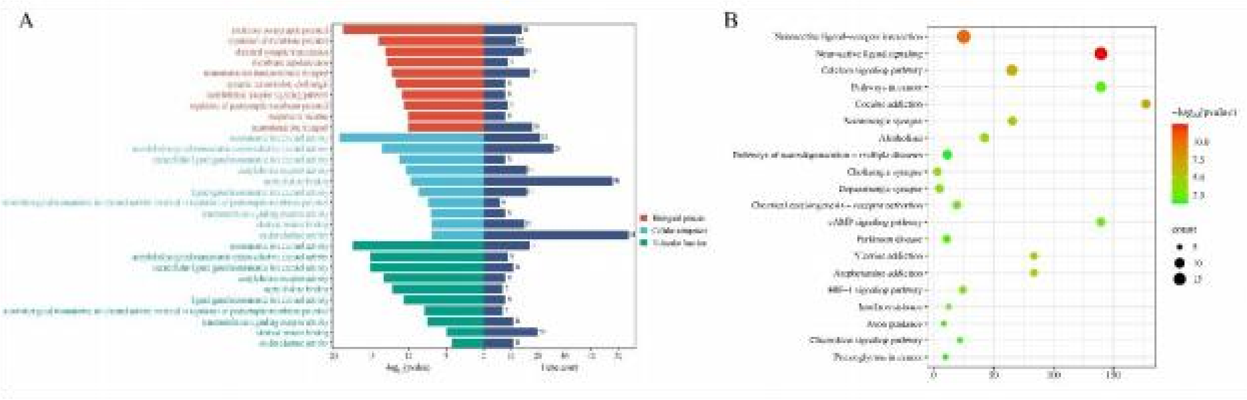

 DOI:10.18332/tid/220849

Figure 7. 2D and 3D molecular docking binding maps of nicotine with depression core targets

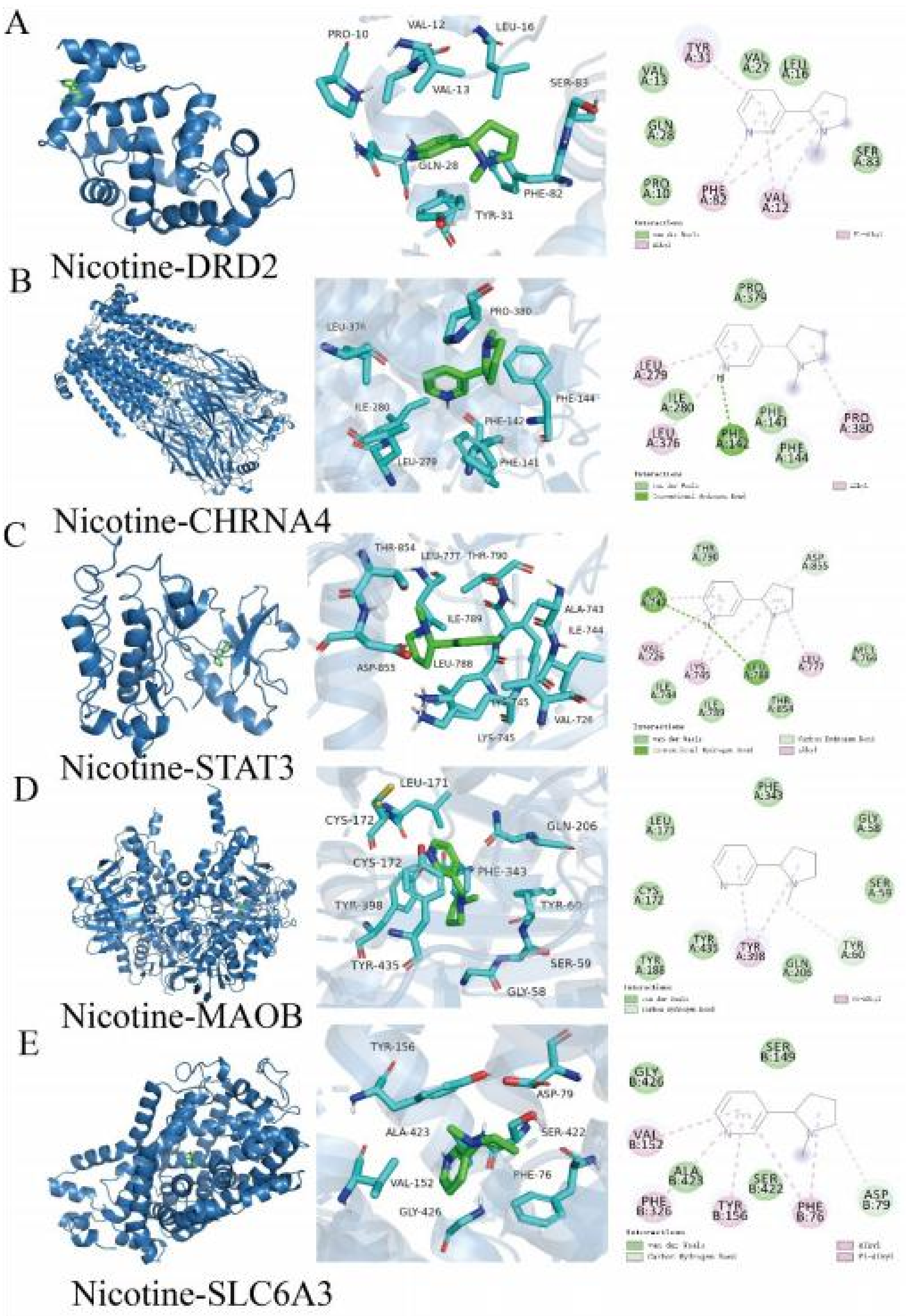

Figure 8. Forest plot of Mendelian randomization analysis for the causal association between 3 core genes and depression risk

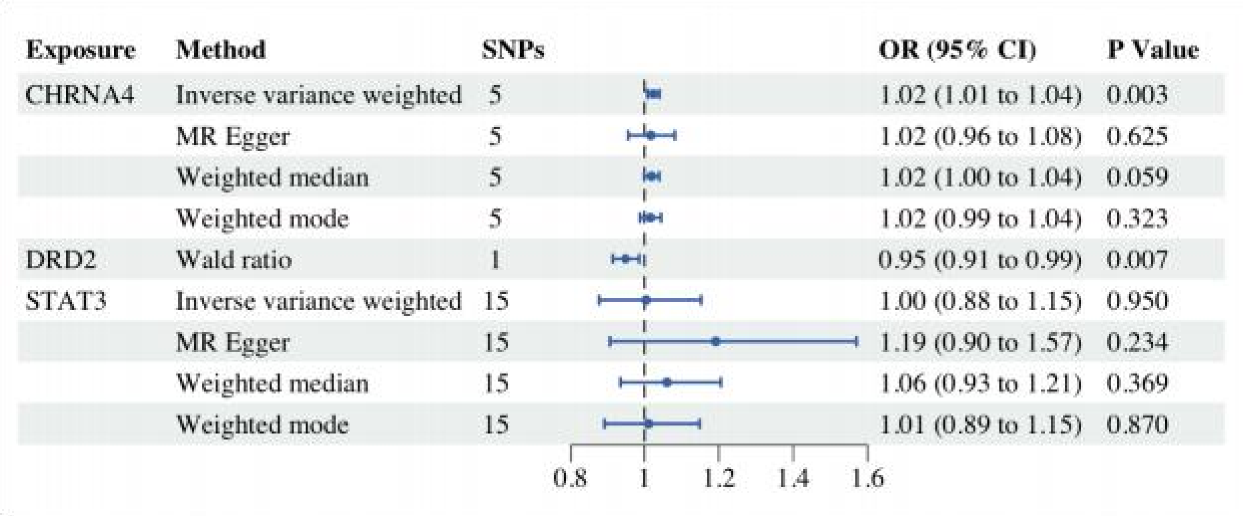

| Exposure | Method | SNPs | OR (95% CI) | P Value |
|---|---|---|---|---|
| CHRNA4 | Inverse variance weighted | 5 | 1.02 (1.01 to 1.04) | 0.003 |
| | MR Egger | 5 | 1.02 (0.96 to 1.08) | 0.625 |
| | Weighted median | 5 | 1.02 (1.00 to 1.04) | 0.059 |
| | Weighted mode | 5 | 1.02 (0.99 to 1.04) | 0.323 |
| DRD2 | Wald ratio | 1 | 0.95 (0.91 to 0.99) | 0.007 |
| STAT3 | Inverse variance weighted | 15 | 1.00 (0.88 to 1.15) | 0.950 |
| | MR Egger | 15 | 1.19 (0.90 to 1.57) | 0.234 |
| | Weighted median | 15 | 1.06 (0.93 to 1.21) | 0.369 |
| | Weighted mode | 15 | 1.01 (0.89 to 1.15) | 0.870 |



© 2026 Li D. et al.

SUPPLEMENTARY FILE DISCLAIMER
The content has been provided by the author(s) and has not been reviewed, verified, or endorsed by European Publishing. It may not have undergone peer review. The views, opinions, and recommendations expressed are solely those of the author(s) and do not necessarily reflect the position of European Publishing. European Publishing accepts no responsibility or liability for any consequences arising from the use of, or reliance on, this content.
